# Developing a Systematic Approach for the Implementation of Medical Extended Reality Learning Modules in Cardiothoracic Health Care: Recommendations From an International Expert Group

**DOI:** 10.1016/j.jacadv.2025.101633

**Published:** 2025-03-26

**Authors:** Edris A.F. Mahtab, Samuel A. Max, Jerry Braun, Madelien V. Regeer, Brian Kaufman, Joel Dunning, Bhuvaneswari Bibleraaj, Martin Andreas, Rafael Rodríguez Lecoq, Milka Klinceva, Rodney Rosalia, Roberto Lorusso, Nico Bruining, Anastasia D. Egorova

**Affiliations:** a Department of Cardiothoracic Surgery, Leiden University Medical Centre, Leiden, the Netherlands; b Department of Cardiothoracic Surgery, Erasmus University Medical Centre, Rotterdam, the Netherlands; c Department of Cardiology, Leiden University Medical Centre, Leiden, the Netherlands; d Departments of Medicine, Anesthesiology & Neurosurgery, New York University Langone Health, New York, New York, USA; e Department of Thoracic Surgery, James Cook University Hospital, Middlesbrough, United Kingdom; f Directorate of Nursing, Midwifery and Social Work Research, University of Salford, Salford, United Kingdom; g Department of Cardiothoracic Surgery, Manchester Foundation Trust, Wythenshawe Hospital, Manchester, United Kingdom; h Medical University of Vienna, Vianna, Austria; i Vall d'Hebron Institute for Research, Barcelona, Spain; j Zan Mitrev Clinic, Skopje, Republic of North Macedonia; k Healthcare Consulting, Skopje, Republic of North Macedonia; l Department of Cardiothoracic Surgery, Maastricht University Medical Center, Maastricht, the Netherlands; m Department of Cardiology, Erasmus University Medical Centre, Rotterdam, the Netherlands; n Centre for Congenital Heart Disease Amsterdam Leiden (CAHAL), Department of Cardiology, Leiden University Medical Centre, Leiden, the Netherlands

**Keywords:** business case, cardiology, cardiothoracic surgery, extended reality (XR), skills simulation, XR training

## Abstract

Extended reality (XR) modalities in health care are quickly evolving. There is a lack of systematically described developmental process and the “how to” execute a business-case guidance. This article formulates a systematic approach on the technical developmental steps and generation of business-case to guide the iterative development of an XR tool. An international expert group was established and several available frameworks related to entrepreneurship and business-case development were used to generate recommendations. Our ongoing experience with the development life cycle of an XR tool for cardiopulmonary resuscitation training is provided as a real-life case illustration. Market demand, value proposition, stakeholder analyses, and profitability scenarios are captured with a business model canvas. Developmental process is divided into 4 aspects: Desirability, Feasibility, Viability, and Scalability. Technical- and Investment Readiness Level models are used in defining the technical feasibility and the business viability and scalability, respectively. Best practice recommendations including examples are provided. Health care professionals, health care financers, and health care policymakers are urged to consider the provided systematic approach and recommendations prior to starting a venture with XR.

## Highlights

• There is a lack of systematically described XR developmental process in health care.
• This manuscript provides a systematic approach and recommendations for development and implementation of XR in health care.
• Health care professionals should consider the provided systematic approach prior to starting a venture with XR.

Numerous extended reality (XR) applications focusing on patients as well as health care professionals as end users have been developed.^1^, ^2^, ^3^, ^4^ The number of scientific research projects with XR applications continues to grow rapidly.^5^ Despite the promising results, XR modalities can encounter several challenges in development and implementation. Creation of haptic feedback, prevention of “cyber sickness,” multimodality image integration, and real-time procedural visualization are examples of technical challenges frequently faced. The majority of the currently available evidence on XR applications in medical field is based on feasibility and observational studies on different modalities and protocols, forming an important source of bias. The exponentially expanding use-cases of XR in health care and clinical education of multidisciplinary teams highlight the urgent necessity for standardization, and guidelines for the development, implementation, and conduct of scientific research in the field of medical XR applications.^1^

Lack of institutional and governmental regulations (laws) makes the development and implementation of these tools uncertain. Three additional major challenges in initiation and implementation of XR projects are significant investments in time and money and lack of technical expertise. Funding limitation on local as well as on (inter)national level for such an innovation compared to clinical trials, medical devices, and/or drugs forms a significant threat for the development of XR modalities in medical field, compared to gaming industry. It is therefore essential to clearly define the project endpoints and identify the potential impact of its successful implementation on current practice at an early stage.

For this paper, an international consortium—VR-SimPort, was established including academic institutions, hospitals, and private sector partners cofunded by the European Union Erasmus Plus Cooperation Partnerships, Leiden University Medical Center Fellowship Grant 2023 as well as Dutch Heart Institute. This article: 1) formulates a systematic approach to technical development of an XR modality for clinical educational use via 9 predefined steps as well as 2) stages for generation of a business-case, and 3) examples for generation of clinical evidence both to be carried out prior to the development and implementation of an XR simulation tool. The established virtual reality (VR)-based cardiopulmonary resuscitation (CPR) simulator (VR-CPR) is used for illustrative purposes throughout the paper (Central Illustration).^6^^,^^7^

**Central Illustration:**
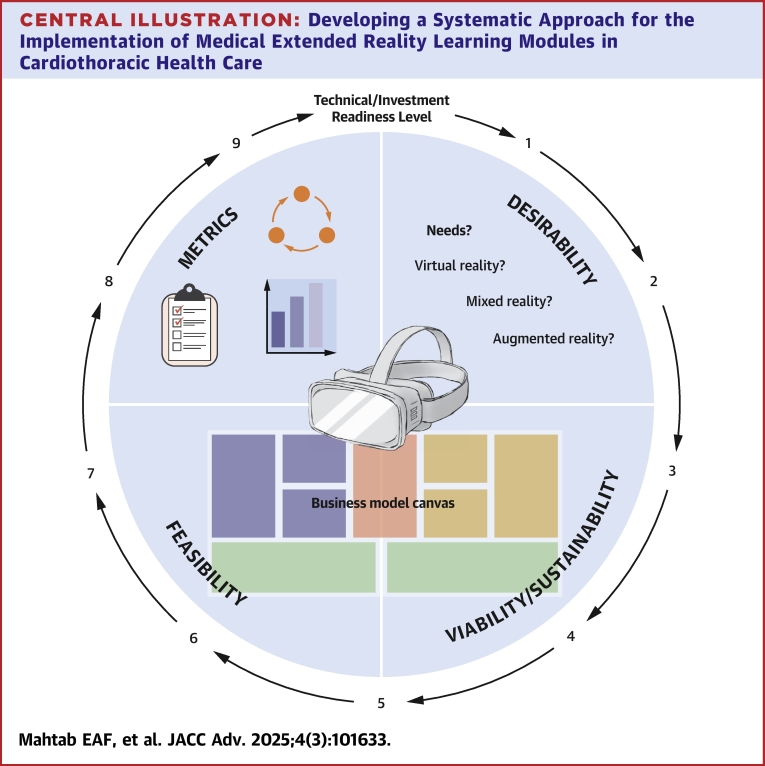
Developing a Systematic Approach for the Implementation of Medical Extended Reality Learning Modules in Cardiothoracic Health Care Prior to implementation of an XR tool, it is important to consider that XR can be broken down in different modalities that constitute this umbrella term. The empathy map^24^ can be used to explore and define the desirability of the target groups. Additional users’ feedback for desirability can be collected using predefined questionnaires.^6^^,^^7^ Feasibility can be explored using TRL model.^9^ Business viability can be systematically evaluated according to IRL model.^25^ The business model canvas (Figure 3)^23^ can be “translated” to the XR field and applied as a basis for planning stages of a new XR tool, where all business segments of this developing tool are analyzed. Final steps in implementation are identification of the relevant metrics^26^ feedback and analysis of the XR tool according to these metrics. XR = extended reality; other abbreviations as in Figure 1.

## Choosing a suitable modality to enhance better implementation

Implementation of digital tools in health care can be challenging. In the field of cardiovascular medicine a position paper on the implementation of digital technology in health care was published by the European Society of Cardiology (ESC) e-Cardiology working group in 2019.^8^ Several barriers and challenges were identified, including technical challenges and the deployment of the right tool. In this perspective, prior to embarking on the development of a specific tool, it is important to consider that XR can be broken down into different modalities that constitute this umbrella term. Each of these submodalities has inherent advantages and disadvantages, and it is important to consider which modalities best fits the scope as defined at the end of the first stage of product definition below.^5^ VR constitutes an entirely virtual 3-dimensional world in which the user is placed, and facilitates the simulation of a location or situation completely distinct from the users’ location when entering the simulation, mixed reality involves the interplay between the physical world and the virtual which is part of it. The virtual scene is influenced by what happens in the physical world, augmented reality involves the user engaging with a virtual scene on top the physical world around them, projected by head-mounted displays or otherwise, whereby the projection of reality does not interact with the physical world. An extensive overview of XR modalities with currently available examples from the daily practice is published by Tsai and colleagues.^2^

## Framework for technical development of XR tools in health care

**Figure 1:**
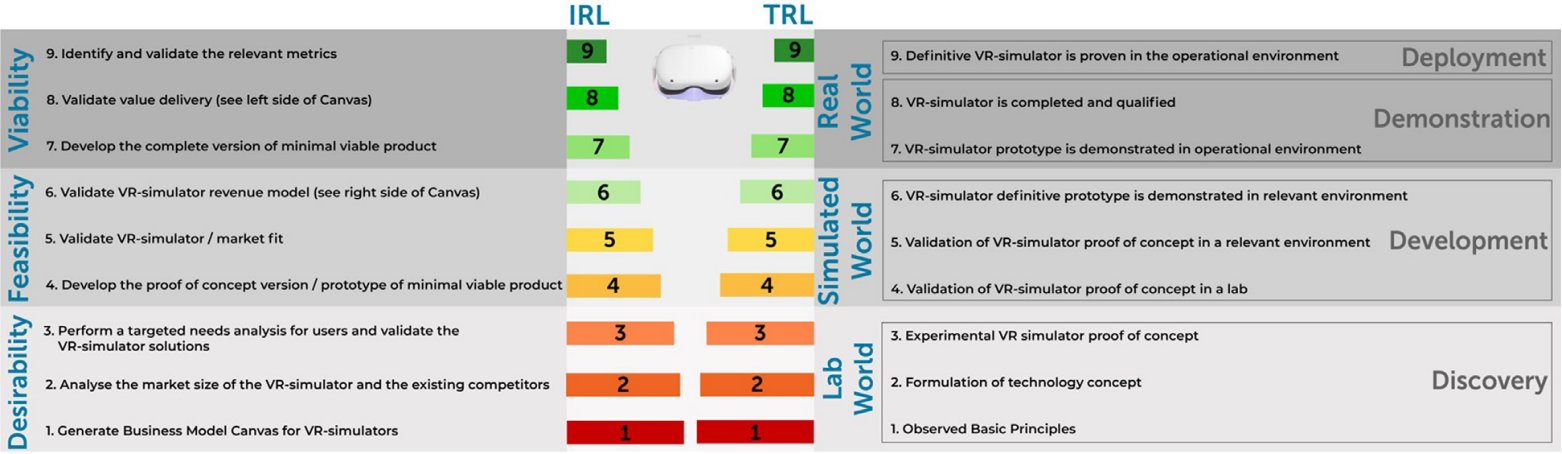
Summary of Recommendations for Technical Readiness Level and Investment Readiness Level for an Extended Reality Module IRL = Investment Readiness Level; VR = virtual reality; TRL = Technology Readiness Level.

The framework that can be used to express the level of XR tools technical development is Technical Readiness Level (TRL) created by The National Aeronautics and Space Administration and adjusted for the public sector^9^ (Figure 1). In this framework, the technical development is systematically defined in 9 sequential steps. In Table 1 for each TRL step an example from the VR-CPR project experience is given.

**Table 1:** VR-CPR Project Experience Examples for Each Technical Readiness Level Step and Business Model Canvas Parts

TRL Stages	VR-CPR Simulator Examples	Business Model Canvas	VR-CPR Simulator Examples
1. Observation and reporting of basic principles	An unmet need for remote training during COVID-19 and difficulties to implement the CPR protocol after cardiac surgery (especially the resternotomy and algorithm learning) for nurses and trainees. Performing a review of the existing solutions as indicated in Table 2.	1. Customer segments	Several target groups for VR-CPR-Sim were defined. The target groups were divided into 3 segments: trainees, trainers, and policymakers. Seven unique user groups were identified as trainees: 1) trainees in cardiothoracic surgery, 2) cardiology and 3) ICU, 4) ward nurses, 5) ICU nurses and 6) CCU nurses, 7) allied health workers including physician assistants, and nurse practitioners. The trainers segment of users consists of medical specialists in the field of cardiothoracic surgery, cardiology, ICU, and ACLS trainers. This segment also includes trainers of nursing and allied health care staff. The last segment of users includes policymakers such as the head of a department, head of training program, ward and ICU managers, financial department, TTO, and institutional legal department.
2. Formulation of technology concept	Surgeons, trainees, and nurses were asked about the XR features that they would deem necessary to be developed. Important aspects were creation of realistic and immersive environment, step-by-step content, easy to use; scenarios based on daily practice and adapted to local as well as (inter) national protocols. Daily patient cases based on CALS/ACLS course and guidelines were used.^14^, ^15^, ^16^, ^17^	2. Value propositions	Value propositions for the trainees in cardiothoracic surgery segment were defined as: Simple, intuitive, and user-friendly interface 1. Algorithm learning with integrated real-life tutorials 2. Realistic clinical situations based on daily practice and protocols 3. Simulator learning: “safe to make mistakes” learning concept 4. Less stress during training and real life clinical situation 5. Faster and more accurate performance 6. Knowledge improvement 7. Realistic 3D environment 8. Single and multiusers modes 9. Standalone and easy software update link, integrated gamming concept (“fun to play”) For illustrative purposes, we focus on cardiothoracic surgery trainees as a case study for this manuscript.
3. Experimental XR simulator proof of concept	An animated movie/presentation was generated and presented to the core development team: a surgeon, 2 surgical trainees, and one clinical software technician. After adjustments of the plan, the first module development was started. Based on the needs and plans, a VR platform was chosen. A USE Questionnaire was adjusted to this project and implemented.^7^	3. Channels	1. Internet website including: a. Helpdesk e-mail address, phone number, postal address b. Information and presentation of the product (including pictures, movies, and scientific proof) c. Section for online purchase of the product d. Newsletters and updates 2. Social media channels providing news, updates, and link to website for purchase and more in-depth information. 3. Symposia, annual meetings, and newsletters of relevant national and international associations (EACTS, ESC). 4. Publication of scientific results in peer-reviewed (inter)national journals. 5. Personal visits with the local contact person 6. Directly approaching key persons and key institutions
4. Testing and validation of XR simulator proof of concept in laboratory environment	The proof of concept was developed first only for ventricular fibrillation scenario where 3 consecutive shocks were delivered. This module was then tested by a surgeon, 2 surgical trainees, one ward nurse (member of hospital CPR team), and one clinical software technician. After adjustments, a pulseless electrical activity scenario was added and the same steps were followed until all scenarios were included.	4. Customer relationships	1. Onsite presentations (at ward, CCU, and ICU) to introduce the VR-CPR-Sim and to start implementation 2. Providing a Helpdesk: email, phone, online website, social media page for troubleshooting and general information 3. Online hands-on sessions for more in-depth tutorial and rehearsal 4. Remote supervision if required 5. Providing a competitive price for this customer 6. Providing Socio-Economic Index-based price 7. Having a dedicated contact person for customer
5. Validation of XR simulator’s proof-of-concept in a relevant environment	A user’s group of surgeons, surgical trainees, and nurses with mixed degree of experience (novel as well as experienced) in CPR was established. The experience of members of the group with VR or gaming was objectified. In this test stage we were interested in how the simulator works and how realistic it was experienced (feasibility). The previously developed USE Questionnaire was used to this aid. For the first time we have demonstrated the simulator to the CALS course representative to check our idea and prevent tunnel vision. The whole simulator was ready. Hereafter, a validity study was executed as described.^7^	5. Key resources	1. Physical a. HMDs, laptops, and other hardware to develop and present the simulator, IT infrastructure, internet connection, and other technical requirements depending on customer b. Hire location/meeting rooms to organize symposia for presentation and dissemination 2. Human a. Software engineers, tutors, physicians/ACLS trainers, business developer, legal advisor, scientific personnel 3. Financial a. Grants (innovation funds, scientific funds) b. Investors, incubators, credits, c. Own capital investments 4. Intellectual a. Educational and medical content b. Inventions idea’s and intellectual properties
6. XR simulator definitive prototype is demonstrated in a relevant environment	The prototype was demonstrated to CPR nurses and CPR tutors from a different department and their feedback was collected. The VR-CPR simulator was compared with the current available educational gold standard training (eg, classroom teaching, hands on training).	6. Key activities	1. Development of real-life scenario’s 2. Development of VR software 3. Development of testing and feedback questionnaires 4. Testing and adjustments 5. Scientific proof (performing studies) 6. Development of PR products 7. Application for relevant certifications
7. XR simulator prototype is demonstrated in operational environment	Simulator was presented to the users from other institutions and a national multicenter RCT was conducted.^6^ Workshops were organized outside the country for feedback and testing in an international setting. Additional needs from the potential users were discovered and implemented (eg, multilingual necessities).	7. Key partnerships	1. Software company/software developers 2. TTO/R&D departments 3. Sponsors (private and governmental) 4. (inter)national professional societies
8. XR simulator is completed and validated	The simulator was demonstrated during the EACTS annual meeting in Vienna in 2023 and several workshops were organized around the globe (Europe, UK, and USA) for dissemination purposes. Relevant posters and promotion material was developed.	8. Cost structure	For the steps 7-9 we have involved the TTO/R&D departments of our institution. As physicians we had not enough expertise and knowledge about the financial regulations, institutional laws, financial laws, intellectual property regulations, etc.
9. Definitive XR simulator is proven in operational environment	The simulator was delivered to the first client at the end of 2023. Users experiences were collected and plans for updates and improvement were generated.	9. Revenue streams	

3D = 3-dimensional; ACLS = acute cardiac life support; CALS = cardiac acute life support; CCU = coronary care unit; CPR = cardiopulmonary resuscitation; EACTS = European Association for Cardiothoracic Surgery; ESC = European Society of Cardiology; HMDs = head-mounted displays; ICU = intensive care unit; R&D = research and development; RCT = randomized controlled trial; TRL = Technical Readiness Level; TTO = transfer technology office; USE = Usefulness, Satisfaction, and Ease of Use; VR = virtual reality; XR = extended reality.

### Observation and reporting of basic principles

The needs and necessities in the current daily practice should be identified via thorough market- and consumer analyses. Qualitative research can be used to explore, collect data, and define these needs, for instance using digital surveys. The need for distanced training, difficulties to implement the existing international guidelines, and the desire to harness the advantages of digitalization in health care education are a few examples of the needs arising from daily practice that can be explored using a digital survey. Lack of personnel, time, finances, and resources pose significant challenges to clinical teaching and learning in daily practice. Finally, a literature review and market analysis for available alternatives should be performed in this step.

### Formulation of technology concept

The foundation of the XR tools technology concept should be established. Key XR features to be considered include the creation of a realistic 3-dimensional environment where required, defining degree of interaction in the virtual world, support for both single and multiuser modes, standalone operation with straightforward software update capabilities, and an integrated gaming concept to enhance engagement (“fun to play”). Scenarios based on real clinical situations and protocols^14^, ^15^, ^16^, ^17^ should be integrated, with a focus on algorithmic learning. The user interface should be simple, intuitive, and user-friendly. Software development should align with protocols of International Organization for Standardization.^10^ Educational methodologies should leverage Blended Learning, Technology-Enhanced Learning, and Simulation-Based Teaching concepts, ensuring a safe environment for making mistakes and learning from this.^11^, ^12^, ^13^ Health care professionals working with the development team should become familiar with the scrum and agile frameworks and use a project management tool such as Jira (Atlassian) that all stakeholders have access to, to centralize, and focus development, with preagreed tasks and milestones optimizing the efficiency of the development process.

### Experimental XR simulator proof of concept

In this phase, development of standardized digital testing and feedback questionnaires should be realized, leveraging existing validated forms including the Usefulness, Satisfaction, and Ease of use Questionnaire,^18^ Technology Acceptance Model, and other related frameworks. Development of a mock-up version and pitching of the proof-of-concept for the purpose of collecting early feedback from stakeholders should be initiated. Any required adjustments of TRL steps 1 to 2 and the proof-of-concept prototype version must then be developed.

### Testing and validation of XR simulator proof of concept in laboratory environment

Testing of the prototype by developers (specifically considering the medical content and technical performance), gathering further user feedback, and making necessary adjustments and refinements of the prototype are core activities in this step. Testing of the adjusted prototype should be continued by a representative panel of potential users (novices and experts) with formal and structured feedback being collected. In this step, testing will usually be performed by workers from own institution/department. However, consulting external collaborators can help avoid “tunnel vision” and ensure the broader applicability of the final simulator. Additional stakeholders including patient advocacy groups and individual patients themselves are additionally advantageous at this stage.

### Validation of XR simulator’s proof-of-concept in a relevant environment

Performing the first face and content validity study with users for whom the XR tool falls within the scope of their practice, with varying experience levels within target group(s).^7^ Focus groups may further benefit targeted development and remediation of early issues. It is important to note that reliability, logistic maintenance, and the ability to update are also relevant points to be under attention. After gathering and processing novel feedback and having started the development of first complete prototype, the minimum viable product (MVP) stage is reached.

### XR simulator definitive prototype is demonstrated in a relevant environment

Testing of the MVP by potential users as in step 5, collecting feedback, and performing adjustments as required. Comparing the MVP with the relevant gold standard of practice, evaluation of the MVP based on its performance, and define and execute adjustments to the tool where required.

### XR simulator prototype is demonstrated in operational environment

Demonstrating and testing the MVP outside of the own institution and collecting feedback to adjust the MVP.

Performing feasibility studies to demonstrate relevant validity criteria and/or a randomized controlled trial, preferably in a multicenter setting, to compare the MVP with a control group who undergo the current gold standard intervention/education.^6^ Apply any new insights and feedback from randomized controlled trial results to improve the product and consider implementing new features and adjustments based on the gathered feedback scored according to the Kano Model^19^ or MoSCoW prioritization method (must-have, should-have, could-have, and won't-have).^20^ Important at this stage is to balance the relative necessity of new features or improvements vs the required development time and/or complexity.

### XR simulator is completed and validated

First version of the definitive product is ready for the users, product is proven and qualified (validated) for the proposed use in daily practice. The relevant certifications (eg, conformité européenne [European conformity], The United States Food and Drug Administration) assessments are applied for the product efficacy and safety, as well as its ability to maintain data safety, reliability, and reproducibility of efficacy. Relevant institutions are approached for cooperation and integration of the XR tool. The product is demonstrated to (inter)national users during symposia and meetings, additional validation studies are performed, in accordance with the recommendations published by Birckhead and colleagues on how to perform scientific studies on XR modules.^21^ A brand identity, and streamlined set of product promotional materials are made available as the product becomes ready for marketing, and buy-in from early stakeholders and clients has been achieved. Customer interface and support strategy are developed to facilitate the dissemination process.

### Definitive XR simulator is proven in operational environment

Product is disseminated to the first clients. Dissemination team is actively approaching new clients, organizing hands-on sessions and online demonstrations. The technical team evaluates the users’ experiences and generates software updates and software troubleshooting. A PDCA cycle (Plan, Do, Check, Act) can be used to evaluate and adjust the product in a proper manner.

## Framework for venture development (viability) of XR tools in the health care

**Figure 2:**
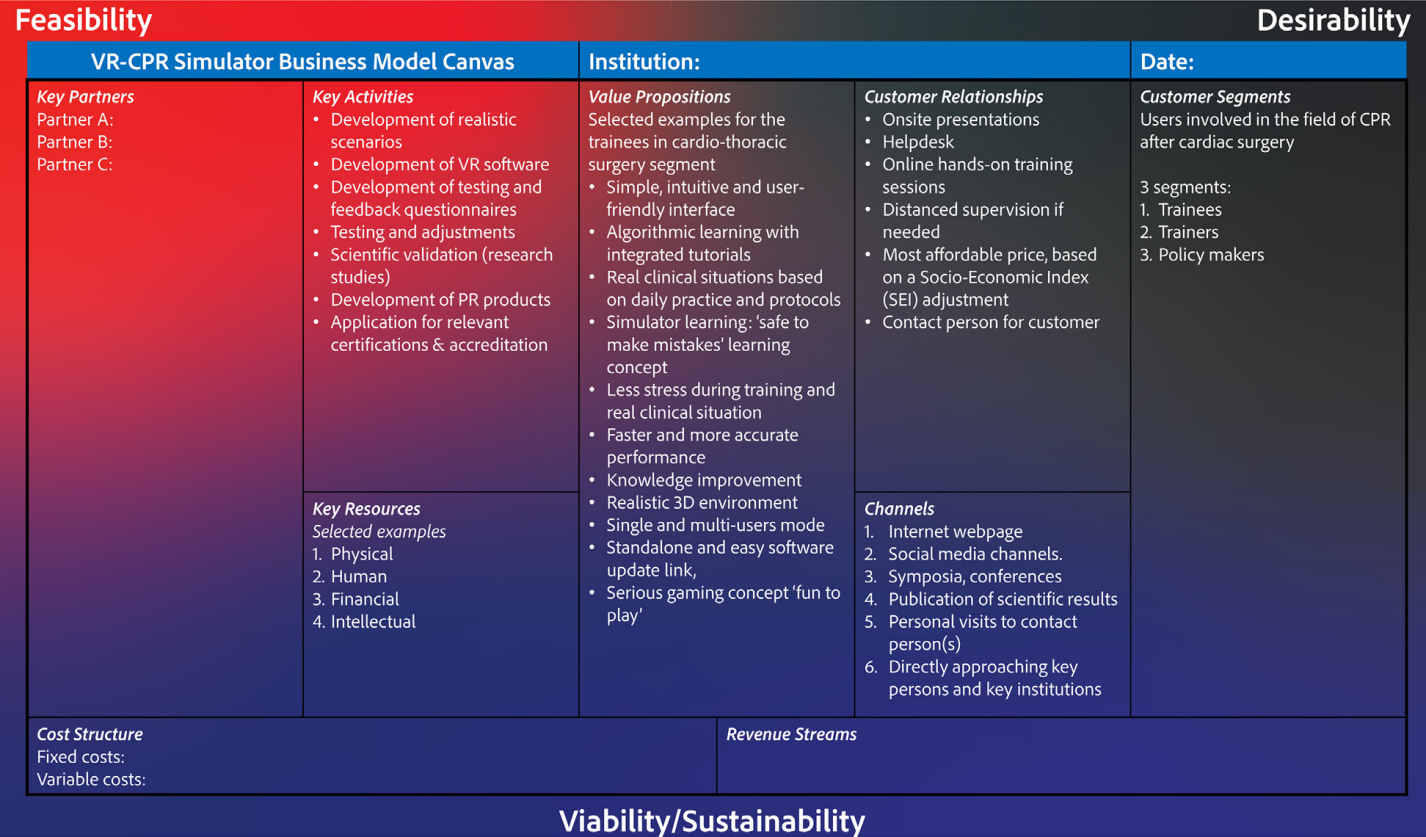
Business Model Canvas Example and Recommendations Summary for an Extended Reality (VR)-Based Cardiopulmonary Resuscitation Simulator (VR-CPR) PR = public relation; CPR = cardiopulmonary resuscitation; 3D = 3-dimensional; other abbreviation as in Figure 1.

In 1995, MacMillan and McGrath established the concept of discovery-driven planning, in which they meticulously described on how to start a new venture.^22^ In 2010, Osterwalder and Pigneur published the handbook “Business Model Generation” where they introduced the business model canvas (Figure 2).^23^ This model can be “translated” to the XR field and applied as a basis for planning stages of a new XR tool, where all business segments of this developing tool are comprehensively analyzed. In this model, the following 4 main key criteria are defined: 1) Desirability, 2) Feasibility, 3) Viability/Sustainability, and 4) Scalability.

The Empathy Map by Dave Gray^24^ can be used to explore and define the desirability of the target groups. Additional users’ feedback for desirability can be collected using predefined questionnaires as described previously.^6^^,^^7^ Feasibility can be explored using TRL model as described above.^9^ Business viability can be systematically evaluated according to Investment Readiness Level (IRL) model described below.^25^

### Business model canvas example of an XR tool

Generate your business model canvas using example given in Figure 2.^23^ This model contains 9 interrelated parts explained below. Health care providers should realize that the valorization process is complex and lies for the most part outside of their daily practice and professional knowledge. Additionally, when health care providers participating in the product development are employed by their (academic) institutions—strict regulations governing intellectual property rights and legal obligations will usually apply. Most importantly, health care providers should identify and appreciate the relation between XR tool content development and potential conflicts of interest in case of valorization, and patent application of the XR product. For these reasons, it is advised to contact the local institutional legal and valorization department for consultation on an early stage of this process. In Table 1, for each business model canvas part, an example from the VR-CPR project experience is provided.

#### Customer segments

The target group(s) should be defined. Note that in the medical setting the term customer might be a different party than the end user. A user is typically the person (of group of professionals) that is actively using the XR solution (eg, trainee) while the customer is the institution (eg, department within the hospital of faculty) that is purchasing the XR solution. This should be taken into consideration when defining the target group. The following 2 questions need to be answered: For which users are you creating this XR tool? Who are the most important customers of this XR tool? Identify customers segment(s) taking into account the following aspects:

1. The need of a customer segment can be different and might require a distinct solution
2. A dedicated segment-specific distribution channel is required to reach each separate segment
3. A diverse relationship is required for each segment
4. Profitability is different for each segment
5. Willingness-to-pay: Distinct market segments may possess divergent factors influencing their purchasing decisions

Examples of customer segments for an XR tool in health care are health care professionals (physicians, nurses, allied health care workers), patients, trainees (students, postgraduate trainees) and trainers, policymakers (managers, department heads, institutional legal department, (inter)national professional societies).

#### Value propositions

The specific value (deliverables) of XR tool to each user segment should be defined using the following questions:

1. Why should this customer group try this XR tool?
2. Which value do you deliver to this specific customer group?
3. Which needs and problems of this specific customer group are addressed by the XR tool?
4. What are the services and (XR) products that you deliver for this specific group?

#### Channels

The goal here is to identify various communication channels to engage with the users effectively. Determine the most efficient methods for achieving several key goals: raising awareness of your XR tool and services, distributing your XR tool to users, offering customer support, facilitating the purchase of your XR tool, and gathering feedback on your XR product and services. The main questions to be answered here are:

1. What are the users’ preferred communication channels for engagement?
2. What are the current primary communication channels for your users? Which ones are you using now?
3. Which channels are most effective in reaching your users?
4. Which channels are cost-effective?
5. How do you integrate the different channels with each other and how do you fit those to your users’ daily practice?

#### Customer relationships

Define the specific relationship for each user segment. Firstly, answer the question: which type of relationship do you have now with a specific user segment? Additionally, determine the possible needs of the user for a relationship (What do they want? How do they want it?). Next, find out what the efficiency of a relationship is with certain user segment and what are the costs? After defining the specific relationships with a specific user segment, integrate it into the business model and workflow.

#### Key resources

Find out the key resources that will facilitate reaching your goals as described in the previous sections: create and present the value proposition, define your users, reach out to them through appropriate channels, establish a relationship with them and reach the market. These key resources can be physical, human, financial, or intellectual. Moreover, these resources are not necessarily in your ownership, as they can also be provided by partners and other third parties.

#### Key activities

Key activities are the most important things to be executed to achieve the goals in defining value proposition, channels, user relationships, and revenue generation. Few examples of key activities in generating an XR tool are:

• Development of realistic scenarios
• Development of XR software
• Selecting appropriate validated questionnaires for testing and feedback
• Testing and adjustments of the XR tool
• Generation of scientific evidence (performing validation studies)
• Development of promotional materials and brand identity
• Application for the relevant certifications (CE, FDA, etc)
• Generating a business model

#### Key partnerships

Partnership can be based on partnership between noncompetitors (strategic), partnerships between competitors (coopetition), and/or joint ventures to develop new businesses.

#### Cost structure

Once the aforementioned sections have been fully defined, the estimated costs associated with the business and development plan can be calculated. One can differentiate between fixed and variable costs that can be applied depending on the type of business model: cost-driven vs value-driven. While the first is focused on a low-cost model, the second model is based on value creation Figure 2.

#### Revenue streams

Before defining the price, market research on the available modalities should be performed. Their current availability, price, and value should be determined. In addition, direct and indirect competitors on the market should be explored. The decision as to whether the price should be different between the previously defined customer segments should be made. Questions like “what is a fair price,” “desired profit,” and “what is the willingness to pay per market segment” should be answered. The implementation of pricing adjusted according to a social development and/or economic index, such as the Socio-Economic Index is an approach that should be considered at this point. This ensures that users from low- and middle-income countries are offered a fair and realistic purchase price for the XR product. It also promotes equitable access by tailoring costs to the economic realities of different countries and regions. Further questions to be answered are: is the price fixed or dynamic, for example, volume- or tier-based? Does the price include the costs of implementation, training, or services such as installation, updates, and customer service? How is the payment organized: automatic billing or periodic invoice based, what are the fees for the client for these facilities, how is the availability of such services regulated on local, regional, national, and international level?

### IRL model example of an XR tool

VR-CPR project experiences: a Master in Business Development professional as well as a qualified external business consultant was involved in reviewing the business plan. The IRL method is applied to define the next venture development steps (Figure 1):

1. Generate business model canvas for XR tool (described above).
2. Analyze the market size of the XR tool and the existing competitors. Define competitors and their characteristics using example Table 2. Please note that competitors can also be outside the XR field: consider web based or mobile phone applications and the conventional tools that meet similar needs.

**Table 2:** Recommendation for Defining Competitors and Their Characteristics During Marketing Search for Available Alternatives in Extended Reality Tool in Health Care

Name	Direct/Indirect Competitor	Location	Founded	Product Category	Focus	XR Technology/Functionality	Business Model	Employees	Revenue
									

**Offices**	**Investors (lead)**	**Funding**	**Public Listing**	**Valuation**	**Partner**	**Endorsements**	**Accreditation**	**Awards**	**Media**

									

XR = extended reality.
3. Define the current needs and problems of the users and validate the XR tool solutions. Select appropriate validated (digital and online) questionnaires to define the needs of the users. Present your proposed XR solution (mock-up version see TRL3) and get feedback from potential users, in order to answer the question: is your XR product a solution to the user’s problem and does it adequately address their needs?
4. Develop the proof-of-concept version/prototype of MVP. See TRL 4 for the development steps.
5. Validate XR simulator and market fit. See TRL 5 for development steps. The MVP is transformed in this step to the Minimal Marketable Product. The primary question for this step is: are there enough users (customers) that want to use your XR product (solution to their problem)? Depending on the *number* of users and on the *frequency* of use, there are 4 categories that can be distinguished:
  a. Few users, low usage frequency (probably not useful to develop this XR product)
  b. Few users, high usage frequency (probably not profitable, however very specific and motivated users segment, further exploration is needed)
  c. Many users, low usage frequency (check the user’s needs, mismatch with needs and solution)
  d. Many users, high usage frequency (your XR solution is a perfect match, probably high profitability)
6. Validate XR simulator revenue model. See Figure 2, the business model canvas. The next question to be answered is: if your XR product is a solution to the problem of the customer (IRL 3), and the customer is willing to use your XR product (IRL 5), then what is the willingness to pay for customers in each segment?
7. Develop the complete version of the MVP. See TRL 5 to 7 for the development steps.
8. Validate value delivery (see left side of the Canvas in Figure 2). In this step, the scalability of the XR tool is evaluated. The question to be answered is: can you deliver the XR tool at a larger scale (upscale for many users)?
9. Identify and validate the relevant metrics. XR product is ready and is brought on the market. Measurement of investment’s profitability is the main goal in this phase. Several marketing metrics are described by Farris et al^26^ that can be used. A few examples include Return on Investment and Customer Acquisition Cost and Customer Lifetime Value Ratio. These metrics are relevant for different stakeholders; it is important to use the relevant metrics for your situation. Products developed through academic innovation development funds with limited equity capital may be scrutinized differently to products developed with mainly external investments.

## Limitations of the frameworks and alternatives

Sharan and colleagues described the use of business models in health care.^27^^,^^28^ The introduction of information technology in health care has accelerated the use of business models and tools in this field. The rise of health-tech startups in health care service delivery and the current limitations has previously been reviewed by Chakraborty and colleagues.^29^ The business model canvas offers a streamlined view of complex realities, acting as a foundational tool that simplifies the intricate nature of business operations. However, given its inherently static and theoretical nature, and to ensure its effectiveness and relevance, the canvas must be complemented by regular updates, adaptations, and the integration of evolving methods and metrics.^30^ This approach allows it to stay reflective of the evolving business landscape, ensuring that the product remains a valuable tool for strategic planning and decision-making. To that end, other tools can be used in conjunction with the business model canvas. To better address this competitive market, one could supplement the business model using Porter's “Five Forces Framework”^31^ with the goal to assess the venture’s resilience amid industrial rivalry. Olechowski and coworkers^32^ described the challenges TRL users face implementing it in the practice. The authors reported 15 challenges allocated in 3 categories: system complexity, planning and review, and validity of assessment. They have also provided solutions that could improve this complex process. Fellnhofer^33^ published an extensive review on the current use of IRL in small and medium-sized companies providing more insight into the different aspects of IRL and alternative approaches.

## Conclusions

The current framework led by an international expert group provides a systematic approach and recommendations based on existing models in the literature for the development and implementation of a XR-based tool in the health care. While applying these models into practice when initiating an XR product development, one should always appreciate the inherent limitations. Health care professionals, financers, and policymakers should consider the provided systematic approach and recommendations prior to starting a venture with XR tools in health care to ensure a streamlined and successful process.

## Funding support and author disclosures

This project was cofunded by the European Union Erasmus Plus Cooperation Partnerships, a Higher Educational Grant, 2022-1-NL01-KA220-HED-000087770 and by the LUMC Research Fellowship Grant 2023. This project is part of the Dutch Heart Institute (NLHI) Working Group on VR-simulation training in acute cardiovascular education. Dr Egorova has received support from the Leiden University Medical Center research council Cardio-Vascular cluster Themes for Innovation funding and the Rembrandt Institute grant; and has received consultancy and speaker fees from Boston Scientific, Medtronic and Abbott. The Department of Cardiology of the LUMC receives unrestricted research grants from Medtronic Trading NL BV, Biotronik Nederland BV, Boston Scientific Nederland BV, Abbott Medical Nederland BV en Edwards Lifesciences LLC. The funders were not involved in study design, collection, analysis, interpretation of data, the writing of this paper, or the decision to submit it for publication. All other authors have reported that they have no relationships relevant to the contents of this paper to disclose.

## References

[1] Mahtab E.A.F., Egorova A.D. (2022). Current and future applications of virtual reality technology for cardiac interventions. Nat Rev Cardiol.

[2] Tsai T.Y., Onuma Y., Zlahoda-Huzior A. (2023). Merging virtual and physical experiences: extended realities in cardiovascular medicine. Eur Heart J.

[3] Sadeghi A.H., Mathari S.E., Abjigitova D. (2022). Current and future applications of virtual, augmented, and mixed reality in cardiothoracic surgery. Ann Thorac Surg.

[4] Sadeghi A.H., Wahadat A.R., Dereci A. (2021). Remote multidisciplinary heart team meetings in immersive virtual reality: a first experience during the COVID-19 pandemic. BMJ Innov.

[5] Kouijzer M.M.T.E., Kip H., Bouman Y.H.A., Kelders S.M. (2023). Implementation of virtual reality in healthcare: a scoping review on the implementation process of virtual reality in various healthcare settings. Implement Sci Commun.

[6] Peek J.J., Max S.A., Bakhuis W. (2023). Virtual reality simulator versus conventional advanced life support training for cardiopulmonary resuscitation post-cardiac surgery: a randomized controlled trial. J Cardiovasc Dev Dis.

[7] Sadeghi A.H., Peek J.J., Max S.A. (2022). Virtual reality simulation training for cardiopulmonary resuscitation after cardiac surgery: face and content validity study. JMIR Serious Games.

[8] Frederix I., Caiani E.G., Dendale P. (2019). ESC e-Cardiology Working Group Position Paper: overcoming challenges in digital health implementation in cardiovascular medicine. Eur J Prev Cardiol.

[9] Héder M. (2017). From NASA to EU: the evolution of the TRL scale in public sector innovation. Innov J.

[10] ISO/IEC/IEEE (2017).

[11] Garrison D.R., Vaughan N.D. (2012).

[12] Ramachandran M. (2017).

[13] Al-Elq A.H. (2010). Simulation-based medical teaching and learning. J Family Community Med.

[14] Brand J., McDonald A., Dunning J. (2018). Management of cardiac arrest following cardiac surgery. BJA Educ.

[15] Dunning J., Fabbri A., Kolh P.H. (2009). Guideline for resuscitation in cardiac arrest after cardiac surgery. Eur J Cardio Thorac Surg.

[16] Dunning J., Levine A., Ley J. (2017). The Society of Thoracic Surgeons expert consensus for the resuscitation of patients who arrest after cardiac surgery. Ann Thorac Surg.

[17] Truhlar A., Deakin C.D., Soar J. (2015). European resuscitation council guidelines for resuscitation 2015: section 4. Cardiac arrest in special circumstances. Resuscitation.

[18] Lund A.M. (2001). Measuring usability with the USE questionnaire. Usability Interface.

[19] Materla T., Cudney E.A., Antony J. (2017). The application of Kano model in the healthcare industry: a systematic literature review. Total Qual Manag Bus Excel.

[20] Kravchenko T., Bogdanova T., Shevgunov T., Silhavy R. (2022).

[21] Birckhead B., Khalil C., Liu X. (2019). Recommendations for methodology of virtual reality clinical trials in health care by an international working group: iterative study. JMIR Ment Health.

[22] MacMillan I., McGrath R. (1995).

[23] Osterwalder A., Pigneur Y. (2010).

[24] Gray D. (2017).

[25] Blank S. (2013).

[26] Paul W., Farris N.T.B., Pfeifer P.E., Reibstein D.J. (2010).

[27] Fredriksson J.J., Mazzocato P., Muhammed R., Savage C. (2017). Business model framework applications in health care: a systematic review. Health Serv Manage Res.

[28] Sharan A.D., Schroeder G.D., West M.E., Vaccaro A.R. (2016). Understanding business models in health care. Clin Spine Surg.

[29] Chakraborty I., Ilavarasan P.V., Edirippulige S. (2021). Health-tech startups in healthcare service delivery: a scoping review. Soc Sci Med.

[30] Vívian Cândido Rodrigues H.E.G.L. (2018).

[31] Porter M.E. (2008).

[32] Olechowski A.L., Eppinger S.D., Joglekar N., Tomaschek K. (2020). Technology readiness levels: shortcomings and improvement opportunities. Systems Eng.

[33] Fellnhofer K. (2015). Literature review: investment readiness level of small and medium sized companies. Int J Manag Financ A.

